# The potential role of exercise in chronic stress-related changes in AMPA receptor phenotype underlying synaptic plasticity

**DOI:** 10.20463/jenb.2017.0037

**Published:** 2017-12-31

**Authors:** Yea-Hyun Leem

**Affiliations:** 1. Department of Human Movement, Seoul Women’s University, Seoul Republic of Korea

**Keywords:** exercise, memory, depression, synaptic plasticity, AMPAR

## Abstract

**[Purpose]:**

Chronic stress can cause disturbances in synaptic plasticity, such as longterm potentiation, along with behavioral defects including memory deficits. One major mechanism sustaining synaptic plasticity involves the dynamics and contents of α-amino-3-hydroxy-5-methyl-4-isoxazolepropionic acid receptors (AMPARs) in the central nervous system. In particular, chronic stress-induced disruption of AMPARs includes it abnormal expression, trafficking, and calcium conductance at glutamatergic synapses, which contributes to synaptic plasticity at excitatory synapses. Exercise has the effect of promoting synaptic plasticity in neurons. However, the contribution of exercise to AMPAR behavior under chronic stressful maladaptation remains unclear.

**[Methods]:**

The present article reviews the information about the chronic stress-related synaptic plasticity and the role of exercise from the previous-published articles.

**[Results]:**

AMPAR-mediated synaptic transmission is an important for chronic stress-related changes of synaptic plasticity, and exercise may at least partly contribute to these episodes.

**[Conclusion]:**

The present article discusses the relationship between AMPARs and synaptic plasticity in chronic stress, as well as the potential role of exercise.

## INTRODUCTION

Stress affects a variety of body systems including the neural, endocrine, immune, and digestive systems. Stress hormones, such as corticosterone, are regulated by the hypothalamic-pituitary-adrenal axis for alertness and adaptation in response to any demand and/or threat ^1^^,^ ^2^. Sustained increases in corticosterone levels result in hippocampal atrophy, impaired long-term potentiation, and reduced neurogenesis, which produces aberrant synaptic plasticity and behavioral abnormalities ^3^^-^^5^. Chronic stress leads to diverse deteriorative consequences in the brain, which in turn impairs cognitive processes, such as learning and memory, and develops into emotion and mood-related illness such as depression ^6^^-^^8^. 

Chronic stress-induced neuronal and behavioral abnormalities are deeply related to synaptic plasticity. Synaptic plasticity refers to the ability of synapses to strengthen or weaken over time in response to altered activity. Aberrant synaptic plasticity, including structural and functional plasticity, emerges under the maladaptive condition evoked by chronic stress ^9^^,^ ^10^. Chronic stress-induced abnormalities in synaptic plasticity are modulated by corticosterone, neurotrophins, oxidative stress, and various neurotransmitters ^8^^,^ ^11^, ^12^^,^^13^. For example, chronic mild stress has been shown to reduce hippocampal transcription of hippocampal brain-derived natriuretic factor (BDNF), which reached basal levels in an isoform-specific manner by KCl-treated depolarization, suggesting that neurotrophins differentially regulate activity-dependent transcription of BDNF ^14^. 

Glutamate, the major excitatory neurotransmitter released from presynaptic terminals, binds to specific receptors that are clustered in the postsynaptic membrane, which mediate the depolarizing signals in glutamatergic synapses. In particular, α-amino-3-hydroxy-5-methyl-4-isoxazolepropionic acid (AMPA) receptors (AMPAR) is a fast ligand-gated cation channel. AMPAR-dependent synaptic influx of cations, especially Ca2+, plays a critical role in synaptic plasticity ^14^^-^^16^. Diverse chronic stress leads to abnormal synaptic function of AMPAR, including AMPAR-mediated excitation in the synapse ^17^^-^^19^. 

Exercise has a beneficial effect on brain functions in both physiological and pathological states. Several studies have suggested that a variety of exercises can enhance hippocampal neurogenesis and neurotransmission underlying the synaptic plasticity related to cognition and mood under normal ^20^^,^ ^21^ and chronic stress conditions ^22^^,^ ^23^. Thus, chronic stress-induced disturbances in synaptic plasticity can result in abnormal neuronal responses and behavioral defects, which can be, at least in part, overcome by exercise intervention. The present review discusses the literature pertaining to the relationship between chronic stress-induced abnormal AMPAR characteristics and synaptic plasticity, as well as the potential role of exercise. 

## The general mechanisms of synaptic plasticity

In general, synaptic plasticity refers to changes in synaptic strength depending on increases or decreases in activity. Synaptic plasticity represents a fundamental mechanism of enabling neurons to generate adaptive responses to stimuli for learning and memory. Subtypes of synaptic plasticity are classified into short-term, long-term, and homeostatic, so-called “synaptic scaling”. 

Short-term synaptic plasticity refers to changes in synaptic efficacy over time, depending on the history of presynaptic activity within hundreds to thousands of microseconds. Short-term depression is produced by neurotransmitter(s) depletion during the synaptic signaling process at the axonal terminal of a pre-synaptic neuron, while short-term facilitation is mediated by Ca2+ influx into the axonal terminal according to spike production, which increases the probability of neurotransmitter(s) release and leads to structural changes (i.e., shape and density) in dendritic spines ^24^. 

Unlike short-term plasticity, long-term plasticity is primarily modulated by gene expression and protein synthesis. Long-term potentiation (LTP), and its counterpart long-term depression (LTD), are two forms of long-term plasticity. Changes at excitatory synapses are long-lasting (i.e., minutes or more). LTD is generated by a minimum level of postsynaptic depolarization as well as increases in the intracellular calcium concentration at the postsynaptic neuron. LTP is the increased synaptic response after the potentiation of prolonged electrical stimuli above the baseline response for hours or longer. Long-lasting synaptic stabilization is regulated by structural changes, including pre- and post-synaptic density, along with the increase in the postsynaptic density protein-95, which causes synaptic enlargement ^25^. 

Homeostatic plasticity refers to the ability of neurons to modify and self-adjust their excitability over a timescale of days. Homeostatic plasticity maintains the stability of neuronal functions through coordinated plasticity among subcellular compartments, such as synapses versus the neurons, and cell bodies versus axons, unlike synapse-specific correlation-based plasticity mechanisms such as LTP and LTD ^26^. 

## The role of the AMPAR in synaptic plasticity

Over the past decades, the molecular mechanisms underpinning synaptic plasticity have been extensively investigated in models of learning and memory. In particular, one of the major mechanisms involved in synaptic plasticity is the dynamics and activity of AMPA-type receptors for controlling plastic changes in the strength and connectivity of glutamatergic or excitatory synapses. 

AMPARs are formed from the tetrameric assembly of subunits GluR1-4, which mediates the fast moment-to-moment transmission of excitatory signals on post-synapses. Endogenous forms of AMPAR primarily consist of GluR1/GluR2 or GluR2/GluR3 heteromers. Glutamatergic synapses that lack AMPAR current― known as “silent synapses”―are not able to achieve sufficient depolarization (excitation) despite containing functional N-methyl-D-aspartate (NMDA) receptors ^27^. In addition to NMDA receptor-dependent Ca2+ influx, AMPAR-dependent synaptic Ca2+ influx is required for NMDA receptor-mediated LTP ^14^^-^^15^^,^ ^28^. Additionally, enhanced LTP expression has been observed in mice with genetic deletion of GluR2 ^29^^,^ ^30^. The phenotypic properties of long-term AMPARs, including synaptic recruitment and calcium permeability, are believed to play a critical role in NMDAR-dependent LTP. These AMPAR dynamics for synaptic plasticity are regulated by its biosynthesis, dendritic transport, exocytosis and endocytosis, through interaction with partner proteins and translational modifications. 

The contents and trafficking of the AMPAR into the plasma membrane through endocytosis and exocytosis is the key regulator of plasticity at glutamatergic synapses ^14^. 

This process is related to Hebbian and homeostatic plasticity, and is implicated in the interaction of several proteins. For example, the association of GluR2/3 with C-kinase 1 contributes to LTP, LTD, and homeostatic plasticity ^31^^,^ ^32^. The binding of GluR2 to N-ethylmaleimide-sensitive factor is also contributes to synaptic incorporation through SNARE-mediated membrane fusion, in which the heteromeric GluR1/GluR2 receptor is recruited into the synaptic site by CaMKII activation ^33^. Recent studies have reported that overexpression of neural precursor cell-expressed developmentally downregulated gene 4-1 (Nedd4-1), a member of the E3 ligase family, reduced the surface density of AMPARs through facilitated endocytosis and the accumulated internalization of GluR1 in the endosome ^34^^,^ ^35^. Downregulation of homeostatic scaling maintains internal excitability via control of synaptic AMPARs content under sustained enhancement of synaptic activity by GABAA receptor antagonism or chronic increased neuronal activity ^35^^,^ ^36^. 

Synaptic insertion of AMPAR into the plasma membrane of excitatory neurons is regulated by protein kinases; PKA- and CaMKII-dependent GluR1 phosphorylation, produces or stabilizes more synapses, thereby controlling synaptic plasticity ^28^^,^ ^37^^,^ ^38^. As mentioned above, the characteristics of AMPAR behavior are an important determinant of synaptic plasticity. 

## Chronic stress and AMPARs under chronic stress-induced maladaptation, and the role of exercise

Chronic stress-induced elevation of glucocorticoid levels affects glutamate transmission and synaptic plasticity, thereby leading to abnormal behavior(s) such as cognitive impairment and depression ^39^. Although, exercise has long been known to improve synaptic plasticity, exercise-elicited AMPAR phenotype alteration in chronic stressful conditions has rarely been investigated. Mounting evidence has demonstrated how chronic stress induces disturbances in AMPAR-dependent synaptic plasticity. For example, chronic stress led to a reduction in AMPAR-dependent excitation of temporoammonic (TA)-CA1 path synapses and a decrease in AMPAR expression of hippocampal CA1 ^17^^,^ ^18^. Chronic restraint stress causes an alteration in AMPAR distribution and function, as well as an increase in neuronal excitatory drive on the basolateral amygdala ^40^^,^ ^41^. Our previous data revealed increased GluR1 content and PKA-directed GluR1 phosphorylation in the basolateral amygdala synapse, along with behavioral depression ^13^. 

Significant evidence supports the exercise-elicited improvement of synaptic plasticity. For example, acute and long-term exercise enhanced the hippocampal expression of synaptic plasticity-related genes, which includes synaptic remodeling-related genes such as synapsin I and synaptotagmin, as well as synaptic plasticity-promoting pathways such as CaMK II and BDNF ^20^. Our previously published study demonstrated that 4-week treadmill running restored chronic stress-induced decreases in hippocampal BDNF expression in an AMPK-dependent manner, along with the reversal of memory impairment ^22^. With regard to the AMPAR, voluntary exercise reversed the decreased field excitatory post-synaptic potential of Schaffer collateral-CA1 pathway concomitantly with enhanced GluR2, which is the less Ca2+-permeable AMPAR assembly, in a genetic rat model of depression ^23^. Furthermore, 4 weeks of voluntary wheel running―but not acute exercise― enhanced GluR1 and pGluR1 (Ser845) levels in the hippocampus ^41^. In unpublished data, exercise exerted an ampakine-like effect on chronic stress-induced failure of memory consolidation and depression-like behaviors, indicated by rendering AMPAR Ca2+ permeable in the CA1 area of the hippocampus. 

Apart from the hippocampus, repeated exercise alters the distribution of AMPAR subunits in diverse brain regions, evidenced by the alteration of AMPAR subunits depending on the duration of sensory-motor cortical area, cerebellum, and striatum ^42^. In the mesolimbic reward pathway, which is closely associated with stress-response plasticity, 6-week running enhanced the expression of tyrosine hydroxylase messenger RNA in the ventral tegmental area and delta opioid receptor in the shell region of the nucleus accumbens ^43^, in which dopamine signaling alters AMPAR-mediated synaptic transmission or potentiation in the nucleus accumbens shell ^44^, suggesting that endogenous dopamine may affect AMPA receptor-mediated Ca2+ conductance. 

As addressed above, chronic stress disrupts AMPAR-mediated synaptic plasticity in some limbic structures, such as the hippocampus, thereby leading to behavioral abnormalities such as cognitive- and mood-related illness. In contrast, exercise may help cope with chronic stress-induced aberrant synaptic plasticity by the incorporation of calcium-permeable AMPAR into the synapse, thereby improving stress-related consequences. 

## Prospective

Chronic stress-induced defects in behavior(s), such as impairment of memory processes and mood-related disorders, are closely linked to synaptic plasticity. This phenomenon has, at least in part, been attributed to the characteristics of AMPARs such as calcium conductance and trafficking. To date, however, the relationship between these electrophysiological and molecular events and exercise has rarely been explored. To clarify this issue, there are several promising prospects. First, we need to investigate what molecules are able to regulate synaptic AMPAR expression, and to determine what signals or partners control AMPAR trafficking in “exercised environments”. Second, to clarify which aspects of an exercise program, including intensity, type, and duration, efficiently affects and/or effects AMPAR-dependent alteration of AMPAR properties. 
